# An Orthopaedic Fracture Clinic Service Audit: A Complete Loop

**DOI:** 10.7759/cureus.1890

**Published:** 2017-11-29

**Authors:** MN Baig, Orna Ni Bhroin, Rajnita Auckloo, Cathal Mac Dhaibheid, Usman Baig, Fergus Byrne

**Affiliations:** 1 Trauma & Orthopaedics, Galway University Hospital; 2 Medicine, Quaid-E-Azam Medical College, Bahawalpur.

**Keywords:** boast guidelines, outpatient department

## Abstract

Introduction

The British Orthopaedic Association Standards for Trauma (BOAST) Guideline 7 informs the standard of care patients should expect when they come to orthopaedic fracture clinics in the United Kingdom (UK).

Objectives

We compared our fracture clinic’s practice against the standards set by BOAST Guideline 7 to make changes for aligning with the standard of care. We aimed to then re-audit our practice for further evaluation against the guidelines.

Material and methods

We prospectively collected data from 100 patients presenting to the fracture clinics of different orthopaedic consultants working in our hospital, using the Royal College of Surgeons in Ireland's (RCSI's) satisfaction with outpatients services (SWOPS) questionnaire. We made some improvements, recommended changes to the hospital management, and conducted a re-audit, collecting data from another 100 patients.

Results

With reference to improvements, we were only able to make them on behalf of the doctors and clinical auxiliary staff. We were able to decrease the waiting time from a patient’s initial presentation in the accident and emergency (A&E) department to an appointment at the fracture clinic. A few improvements were made to the waiting area facilities. However, the cumulative changes resulted in a positive attitude in patient satisfaction levels.

Conclusion

Considering our complete audit loop, we found gaps and enabled improvements, but areas of concern remain, which will need to be addressed in the future.

## Introduction

Fracture clinics are a major part of any trauma and orthopaedic service. These are clinics in which orthopaedic doctors see and manage patients with acute bony or soft tissue injuries. Fracture clinics syphon a large part of human and material resources from the trauma and orthopaedic departments and, eventually, from the hospital in general. It is evident that patients visiting the fracture clinics have a recent acute injury affecting their lifestyle and daily living significantly [1].

The first guidelines for fracture clinics were introduced by the British Medical Association in 1935 to prevent the mismanagement of bony or soft tissue injuries and to standardize patient care. The most recent guidelines are the British Orthopaedic Association Standards for Trauma (BOAST) Guideline 7, released in August 2013 [2]. Our hospital is a tertiary care facility in the West of Ireland with a potential patient population of approximately one million people. On average, we see about 24,000 patients in our clinics; the majority of them are fracture clinic patients. We conducted an audit surveying our outpatient fracture clinic practices and facilities to compare them against the BOAST 7 guidelines. We introduced some changes, and then conducted the second leg of the audit, thus completing the loop. This report shares the results of the audit and discusses our practice, shortcomings, and improvements and their outcomes. Informed consent was obtained from the patients for this study.

## Materials and methods

**Figure 1:**
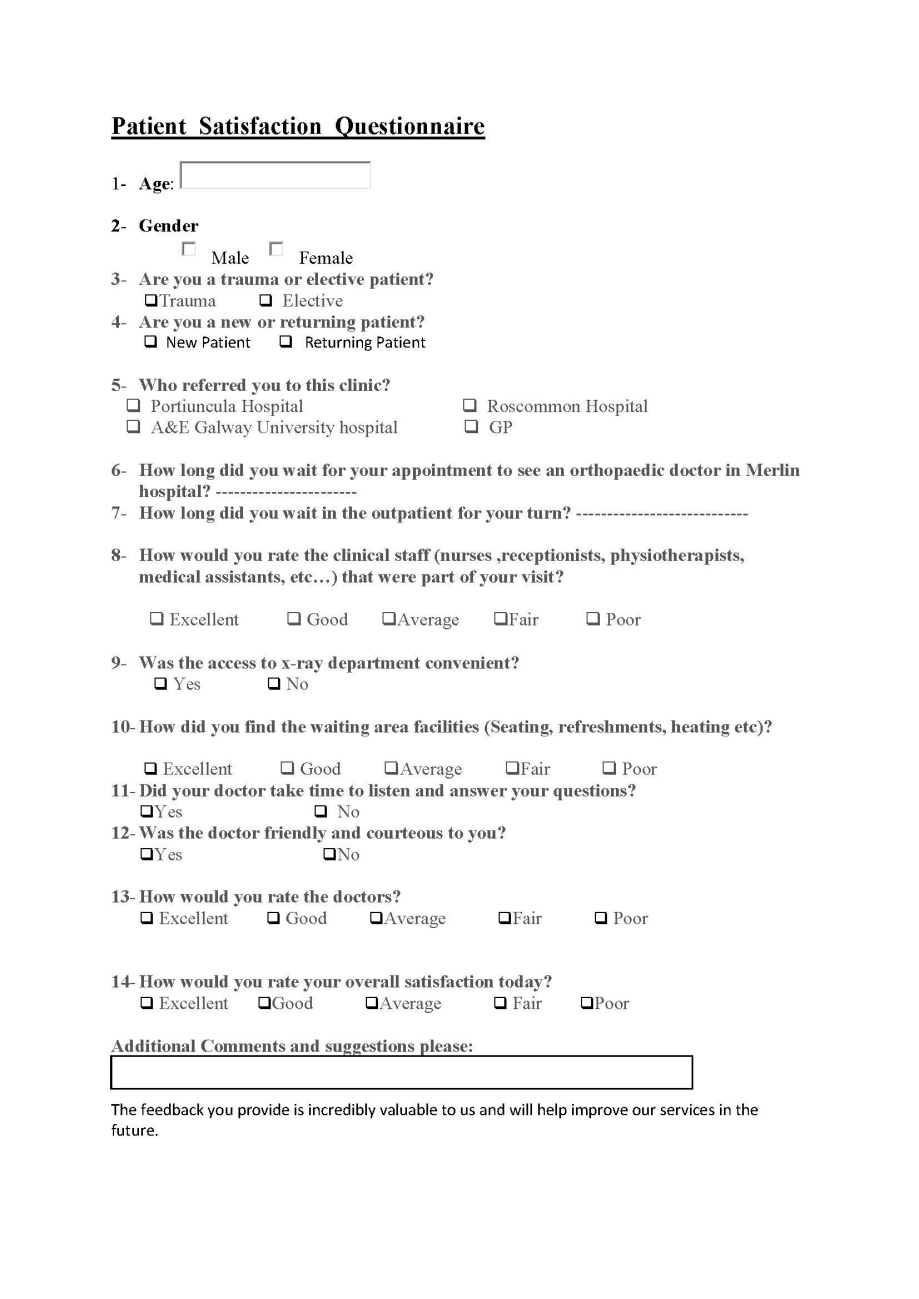
Patient satisfaction questionnaire Modified SWOPS questionnaire SWOPS: satisfaction with outpatients services

We developed a questionnaire based on the Royal College of Surgeons Ireland’s (RCSI's) satisfaction with outpatients services (SWOPS) questionnaire [3]. We customised the questionnaire according to the BOAST Guidelines (Figure 1). We collected the data from 100 patients coming to our fracture clinic, with no exclusion criteria. The key points in the questionnaire were the waiting time for the clinic appointment/to see the doctor in the clinic, the rating of the doctors and clinical staff, and the rating of the facilities (e.g., distance to the x-ray area/patient waiting area).

Our standard of comparison was BOAST Guideline 7 [2]. The most significant issue was the duration of time it takes the patients to be seen in an orthopaedic clinic, starting from their presentation in the accident and emergency (A&E) department. The second area of concern was the inadequate facilities in the waiting area and the walking distance to the x-ray department. The rating of the doctors and clinical staff was quite encouraging. The only repeated concern was that patients wanted to be seen by the previous doctor they saw on their last visit or on their return from their x-ray in the clinic; patients desired a feeling of continuous care.

## Results

We made some changes in the appointment rotation of the fracture clinic to decrease patient waiting times. We also made sure that, at least, when the patient came back from an x-ray, they were seen by the same doctor. This satisfied the patient-reported need for continuous care. The waiting area facilities were improved but not as well as we would have hoped. We increased the seating capacity to some extent. The distance of the x-ray department was another issue of patient dissatisfaction. The results of the pre-change audit and the post-change audit are shown in Table 1.

**Table 1:** Audit results Results in different categories

Parameters N=100	Pre-Changes (July-Aug) N=100	Post-Changes (Sept-Oct) N=100
Age (Average)	41.86 years	45.5 years
Gender	Male: 50.72%; Female: 49.28%	Male: 52.45%, Female: 47.55%
Waiting Time for Appointment (Average)	3.55 days (85 hours)	3.21 (77.04 hours)
Waiting Time to See Doctor in OPD	56.81 minutes	52.55 minutes
Clinical Staff Ratings (Nurses, physio, plaster technicians, etc.)	Excellent = 68.11%; Good = 24.63%; Average = 4.3%; Fair = 2.8%; Poor = 0	Excellent = 69.5%; Good = 24.63%; Average = 2.8%; Fair = 2.8%; Poor = 0
Doctor Ratings	Excellent = 75%; Good = 21.73%; Average = 2.8%; Fair = 0; Poor = 0	Excellent = 73.9%; Good = 24.63%; Average = 0; Fair = 1.4%; Poor = 0
X-ray Facilities Rating	Adequate = 65.21% Inadequate = 34.7%	Adequate = 67.21% Inadequate = 32.79%
Waiting Area Ratings	Adequate = 55.07% Inadequate = 44.93%	Adequate = 58.51% Inadequate = 41.49%
Overall Experience	Excellent = 50.72%; Good = 31.8%; Average = 11.59%; Fair = 4.34%; Poor = 1.44%	Excellent = 53.55%; Good = 31.0%; Average = 13.56%; Fair = 1.87%; Poor = 0

## Discussion

**Figure 2:**
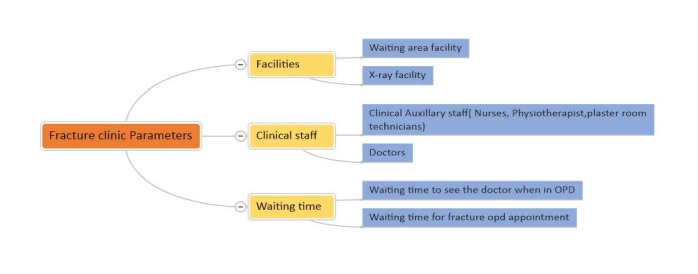
Broad area of the audit Three main stems of the audit and sub-divisions

It is important to complete an audit loop to evaluate the services provided and realize areas needing improvement [4]. Our clinics are consultant-led, with physiotherapy/plaster room facilities available under the same roof, and x-ray, computed tomography, and magnetic resonance imaging available in the close vicinity. Patient medical records and previous and current images are readily available. Planned admission facilities and osteoporotic clinic liaison arrangements are also available. In our consultant-led clinics, the management plans are made by the registrars and the consultant. These services satisfy most of the BOAST 7 guidelines. We divided our audit into three broader areas, as shown in Figure 2.

For waiting times, we were able to improve the waiting time of the patient’s first appointment from 3.5 days (85 hours) to 3.21 (77.2 hours). This decline in waiting times may have been affected by a change in weather; patient attendance tends to decline as cold winter weather arrives. A large number of patients remains a challenge for further wait time reduction. We were also able to improve waiting time to see the doctor inside the clinic from 56.8 minutes to 55.2 minutes. While this change is not a large improvement, it is a step in the right direction. We increased the number of doctors on site and optimized the process of sending patients for follow-up x-rays.

The audit also evaluated the resourcefulness, helpfulness, and level of courtesy of the doctors and the clinical staff. Doctors scored a 75% excellent rating in the first stage of the audit and a 74% excellent rating in the second stage of the audit. For our clinical staff (which includes nurses, receptionists, physiotherapists and plaster technicians), the excellence rating was 68% in the first stage and 69% in the second stage. Although decent, there is always room for further improvement.

The third area of interest was the clinic’s facilities, including the x-ray facility and the patient waiting area. These were areas where many patients were not satisfied. The main problem with the x-ray facility is its location at a five-minute walk from the clinic itself, with part of the path that patients must travel being outdoors, where patients are exposed to inclement weather. This is especially troublesome for elderly patients. We provided our findings and suggestions to the hospital management. Given that moving the x-ray facility to a more convenient location requires a large amount of planning and resources, the move remains a long-term goal.

Our waiting area has a seating capacity of approximately 40 patients. Usually, we have one or more attendants with the patients, but the number of attendants available is not sufficient to meet patient needs. With the help of the management, we have increased the waiting area seating size to accommodate 50 patients, but more work is needed to improve the rating for the waiting facility. 

## Conclusions

This audit loop shows that patient satisfaction not only depends on being treated and being seen by the doctor but multiple factors, including waiting area, x-ray facility, waiting time for the appointment and waiting time in the clinic, contribute to patient satisfaction. This audit loop shows that although we have made some improvement by changing our practice and improving facilities, many things can be done to further improve the fracture clinic’s facilities.
